# Diagnostic Accuracy of Cerebroplacental Ratio in Prediction of Postnatal Outcomes in Oligohydramnios

**DOI:** 10.7759/cureus.33131

**Published:** 2022-12-30

**Authors:** Huma Mahmood Mughal, Mahjabeen Mahmood Kamal, Hammad Ayaz, Muhammad Wasim Awan, Naila Nasir Usmani, Shaghaf Iqbal, Maham Bilal, Abu Bakar Niazi, Hassan Mumtaz

**Affiliations:** 1 Radiology, Khan Research Laboratories (KRL) Hospital, Islamabad, PAK; 2 Diagnostic Radiology, Khan Research Laboratories (KRL) Hospital, Islamabad, PAK; 3 Radiology, Shifa International Hospital, Islamabad, PAK; 4 Medicine, Dow University of Health Sciences, Civil Hospital Karachi, Karachi, PAK; 5 Medicine, Mayo Hospital, Lahore, PAK; 6 Urology, Guy's and St. Thomas' Hospital, London, GBR; 7 General Practice, Surrey Docks Health Centre, London, GBR; 8 Public Health, Health Services Academy, Islamabad, PAK; 9 Clinical Research Center, Maroof International Hospital, Islamabad, PAK

**Keywords:** fetal doppler, postnatal outcome, cerebroplacental ratio, isolated oligohydramnios, cpr

## Abstract

Background and aim: The evidence on isolated oligohydramnios (IO) patients and their postnatal outcomes are inconsistent. Recent research has clarified the connection between that IO and negative outcomes in the postnatal period. Our goal was to analyze the correlation between Doppler measurements and postnatal outcomes in oligohydramnios patients, with a focus on the cerebroplacental ratio (CPR).

Methodology: A cohort study was conducted in the Radiology Department of Khan Research Laboratories (KRL) Hospital from October 2021 to July 2022. One hundred women were chosen as the sample size. For this study, we used the Raosoft sample size calculator with a 95% confidence interval and a 5% margin of error. Both the middle cerebral artery and the umbilical artery were imaged using ultrasound, and the systolic-to-diastolic ratio and peak systolic velocity are recorded. Pulsatility index (PI) and resistive index (RI) were also calculated. If the amniotic fluid index (AFI) is less than 5 cm, the condition is known as oligohydramnios. The newborn's APGAR score was taken immediately after birth as well as after 5 minutes.

Results: We have determined that, on average, mothers are 35.45 weeks/248.15 days pregnant. When compared to the reference standard, CPR diagnostic features showed a sensitivity of 92% and a specificity of 77.27. Overall diagnostic accuracy is predicted to be 93.0%, with a 93.50% positive prognosis and a 73.91% negative prognosis. The effect size for the change in APGAR scores before and after the test was -2.38 1.03, with a 95% confidence interval of -2.58 to -2.17 and a significance level of 0.00.

Conclusion: This study concludes that CPR is an effective screening tool and that it can be used to predict postnatal outcomes in patients with oligohydramnios. Clinical prediction rules were found to be a more effective screening tool, with a sensitivity of 92%, a specificity of 77.27%, and a diagnostic accuracy of 92.3%.

## Introduction

Reduced amniotic fluid volume during pregnancy is medically referred to as oligohydramnios. The occurrence rate is low (0.5%) yet nonetheless significant (5%). Patient population, gestational age, and assessment techniques are all contributors to its prevalence. Oligohydramnios can be diagnosed with an amniotic fluid index of less than 5 cm and a single deepest pocket of less than 2 cm [1].

The health and growth of the fetus depend on a constant supply of amniotic fluid. Additionally, amniotic fluid aids in shielding the developing baby from harm. Oligohydramnios can be caused by multiple fetal factors, including placental malfunction, chromosomal abnormalities, medication adverse effects, and premature membrane rupture. Consequences include a higher rate of cesarean sections, meconium aspiration syndrome, critical care unit admission, and low APGAR score in newborns [2]. The results of the routine antenatal imaging with ultrasound (RADIUS) experiment, which was done on patients with isolated oligohydramnios (IO), showed that fetal growth was unaffected. However, even if the growth percentile is just at the 10% level, placental insufficiency is a possibility [3]. Furthermore, 13.2% of IO pregnancies with unrecognized fetal growth retardation have been documented in some studies [4].

There is a lack of evidence for inducing labor in IO, as stated by the American College of Obstetricians and Gynecologists (ACOG) in 1999. Consequently, there is no definitive answer about the optimal time of birth for these individuals according to ACOG's position [5]. Despite this knowledge, doctors may nevertheless decide to induce birth in these women [6]. Amniotic fluid, fetal vascular structures, maternal uterine circulation, and Doppler of the umbilical artery and ductus venosus are commonly utilized to assess fetal development and health.

Nowadays, CPR is utilized to show how much blood is being sent to the brain. The enlargement of blood vessels in the brain is to blame for this. Diastolic flow and placental bed resistance are both lowered during intrauterine growth restriction (IUGR). The index of middle cerebral artery (MCA) resistance is calculated by dividing the index of the umbilical artery (UA) by the pulsatility index (PI). Compared to MCA and UA Doppler, measuring CPR is a more accurate diagnostic tool for fetal growth limitation [7]. Changes in estimated fetal weight might make fetal growth restriction (FGR) difficult to diagnose. Performing CPR could be an effective method of monitoring fetal hypoxia in such situations [8].

Inconsistencies in the results of literature searches for the management of pregnancies with isolated oligohydramnios (IO) suggest a knowledge gap among clinicians. There is a strong correlation between IO and negative delivery outcomes. However, there is no clear solution to the question of when these women should give birth. The major goal of this study was to examine the association between Doppler measurements (CPR) and perinatal outcomes in oligohydramnios patients.

## Materials and methods

This cohort study was conducted in the Department of Radiology, Khan Research Laboratories (KRL) Hospital Islamabad, from October 2021 to July 2022. One hundred women were chosen as the sample size. The margin of error was set at 5%, and a 95% confidence interval was maintained using a software sample size calculator. We included all pregnant women who presented to the labor and delivery ward with oligohydramnios. Women with multiple fetuses, gestational hypertension, preeclampsia, diabetes, premature birth, chromosomal abnormalities, structural abnormalities, cholestasis, eclampsia, renal, or cardiac problems were not included. KRL Hospital's Institutional Ethical Review Board approval was obtained before starting data collection. Informed written consent was obtained from all subjects prior to participation.

Participants were examined using ultrasonography by a consultant radiologist having at least 10 years of experience. Both the middle cerebral artery and the umbilical artery are imaged using ultrasound, and the systolic-to-diastolic ratio and peak systolic velocity are recorded. If the amniotic fluid index (AFI) is less than 5 cm, the condition is known as oligohydramnios. The newborn's APGAR score was taken immediately after birth as well as after 5 minutes. The formula for the resistance index (RI) is as follows: RI = (peak systolic velocity - end-diastolic velocity)/PSV mean systolic pressure = mean diastolic pressure x end-diastolic pressure. Peak systolic velocity minus end diastolic velocity divided by time-averaged velocity is the formula for pulsatility index (PI).

Data analysis

Statistics were performed using SPSS version 21.0 (Armonk, NY: IBM Corp.). The correlation was analyzed using the chi-square test. The t-test was used to analyze the change in APGAR scores between the two time periods. We calculated the positive and negative correlation between two variables using a two-by-two table. Accuracy in both prognosis and diagnosis, using the APGAR score as a reference point. According to a 2016 study, a CPR of less than 1.0 was regarded to be negative, whereas a CPR of greater than 1.0 was considered to be positive [9].

## Results

With a gestational age between 32 and 35 weeks, 23 (51.91%) of the females were G2p1 and four (8.33%) were G8P7. At 36-38 week gestation, 13 (25%) females were G2p1 and seven (13.46%) were G8P7 having a significant p-value of 0.003 (Table 1).

**Table 1:** Correlation of gestational age with gravidity. G: gravida; P: para

Gestational age	Gravidity				p-Value
	G2P1	G4P3	G6P5	G8P7	0.003
32-35 weeks	23	9	12	4	
36-38 weeks	13	21	11	7	

In women with pregnancy of 32-35 weeks, 75% of women had a Doppler PI for the umbilical artery that was less than 1, while 25% of patients had a PI of greater than 1. Table 2 demonstrates that during 36 and 38 weeks of pregnancy, RI was higher in 23 participants (45.09%), having a significant p-value of 0.001. Umbilical artery Doppler RI was lower than 1 in 48 females among women 32-35 weeks pregnant. Table 3 shows that at weeks 36-38 of pregnancy, 12 people had RI greater than 1, having a significant p-value of 0.000.

**Table 2:** Correlation of gestational age with umbilical artery Doppler PI. PI: pulsatility index

Gestational age	Umbilical artery Doppler PI		p-Value
	Less than 1.0	More than 1.0	0.001
32-35 weeks	36	12	
36-38 weeks	28	23	

**Table 3:** Correlation of gestational age with umbilical artery Doppler RI. RI: resistive index

Gestational age	Umbilical artery Doppler RI		p-Value
	Less than 1.0	More than 1.0	
32-35 weeks	48	0	0.000
36-38 weeks	12	40	

There were 11 (23.91%) patients having less than 1.0 PI and more than 1.0 PI in 35 (76.09%) patients among pregnant women who were 32-35 weeks pregnant. Table 4 shows that during 36 and 38 weeks of pregnancy, fewer than 1.0 PI occurred in 4 (7.3%) and more than 1 PI occurred in 48 (92.3%) instances, having a significant p-value of 0.000.

**Table 4:** Correlation of gestational age with middle cerebral artery Doppler PI. PI: pulsatility index

Gestational age	Middle cerebral artery Doppler PI		p-Value
	Less than 1.0	More than 1.0	0.000
32-35 weeks	11	35	
36-38 weeks	4	48	

Among women between 32 and 35 weeks of pregnancy, middle cerebral artery Doppler RI was less than 0.50 in 12 (25%) and greater than 0.50 in 36 (75%) cases. During 36-38 weeks of gestation, 52 individuals had RI higher than 0.50 having a significant p-value of 0.000 (Table 5). An effect size of -2.38±1.03 was observed between the pre- and post-test APGAR scores, with a 95% confidence interval of -2.58 to -2.17 having a p-value of 0.000 (Table 6).

**Table 5:** Correlation of gestational age with middle cerebral artery Doppler RI. RI: resistive index

Gestational age	Middle cerebral artery Doppler RI		p-Value
	<0.50	>0.50	0.000
32-35 weeks	12	36	
36-38 weeks	0	52	

**Table 6:** Pre- and post-APGAR score effect.

	Mean	SD	Standard error of mean	95% CI of the difference	p-Value
Pre- and post-APGAR	-2.38000	1.03260	0.10326	-2.58489 to -2.17511	0.000

When compared to the gold standard, the diagnostic parameters of CPR showed a sensitivity of 92% and a specificity of 77.27%. Prognostic accuracy was 93.50% positive, 73.91% negative, and diagnostic accuracy was 92.3% (Table 7).

**Table 7:** Diagnostic parameters of CPR compared to the gold standard. CPR: cerebroplacental ratio

APGAR score	CPR	
	Positive	Negative
Positive	72	5
Negative	6	17

## Discussion

With this cohort study, we want to evaluate the diagnostic accuracy of CPR in determining the postnatal outcome of patients with oligohydramnios. Patients with low CPR are more likely to have cesarean sections, have worse 1- and 5-point APGAR ratings, and be admitted to the neonatal intensive care unit. There has been some debate about the long-term effects of oligohydramnios on the health of the fetus, based on earlier research. The 2013 meta-analysis found no substantial variations in postnatal outcomes associated with oligohydramnios. While APGAR scores, NICU admission rates, mortality rates, and umbilical artery pH are all unaffected by the rise in cesarean sections, there is an increase in cesarean sections overall [10].

Meconium-stained amniotic fluid was also not linked to oligohydramnios [10]. According to the findings of another study, the optimal time of delivery was found for patients with oligohydramnios [11]. Admittance to the NICU, low umbilical cord pH, and low APGAR ratings were neither correlated nor dissimilar [11]. In 2016, researchers published a meta-analysis that found a link between IO and unfavorable newborn outcomes. Similar to our findings, other studies have found that nonreassuring fetal heart rate (NRFHR) in oligohydramnios individuals is associated with a higher risk of cesarean delivery, a higher likelihood of NICU admission, and low 1st and 5th APGAR scores in IO patients [12].

In 2017, researchers performed another meta-analysis that confirmed the link between oligohydramnios, low APGAR scores, and cesarean delivery rates. Meconium aspiration syndrome was also discovered to be linked, which we did not find in our analysis [13]. Due to a lack of a large enough sample size, our study cannot draw any conclusions about a possible link between IO and stillbirth. The 2017 study by Khalil et al. demonstrated the link between low CPR and poor fetal growth and newborn outcomes [14]. It was also determined that a low CPR can be utilized as a biometric marker for estimating a person's pace of growth. Several studies have also discovered a correlation between inadequate CPR and the health of the developing baby [13,14].

In a study conducted at the University of Ghana Medical School, Accra, Ghana, fetuses with a CPR of less than 1.1 were considered to be at risk. Composite unfavorable perinatal outcomes could be predicted with a sensitivity of 29.4% and a specificity of 100% when low CPR (1.1) was present, which was contrary to our findings [15].

Consistent with our findings, a study from 2017 at Lady Willingdon Hospital, Lahore, found that the diagnostic accuracy, specificity, sensitivity, positive predictive value, and negative predictive value of the CPR for low APGAR score were 86.94%, 97.18%, 84.21%, 97.64%, respectively [16]. The cerebroplacental ratio has the highest sensitivity in predicting fetal heart rate anomalies and unfavorable newborn outcomes in term, uncomplicated pregnancies, according to research from Suez Canal University in Ismailia, Egypt [17].

The risk of neonatal morbidity was however not predicted by the umbilical artery Doppler or cerebroplacental ratio for severe or borderline oligohydramnios, according to a systematic review and cost-effectiveness analysis conducted by the National Institute for Health Research (NIHR) at Cambridge University, United Kingdom. Existing research suggests that ubiquitous ultrasonography for fetal presentation solely may be clinically and economically justified. Given the state of our knowledge, it is possible that universal screening for presentation at term is warranted [18].

Limitations

However, we could only present our findings narratively due to the inherent limitations in the included studies, such as large heterogeneity in the study populations, inconsistencies in the definition of pregnancy outcomes, differences in the gestational age at the Doppler study, and differences in the prognostic accuracy measures reported. It is suggested that in the future, a comprehensive review of relevant literature is conducted and a meta-analysis is performed.

## Conclusions

In this research, CPR's screening efficacy and ability to predict postnatal outcomes in patients with oligohydramnios were examined. Clinical prediction rules were found to be a more effective screening tool, with a sensitivity of 92%, a specificity of 77.27%, and a diagnostic accuracy of 92.3%. Clinicians are advised to closely monitor IO patients with low CPR values to ascertain the likelihood of adverse neonatal outcomes and fetal discomfort.
